# Inhibition of Expression in *Escherichia coli* of a Virulence Regulator MglB of *Francisella tularensis* Using External Guide Sequence Technology

**DOI:** 10.1371/journal.pone.0003719

**Published:** 2008-11-13

**Authors:** Gaoping Xiao, Eirik W. Lundblad, Mina Izadjoo, Sidney Altman

**Affiliations:** 1 Department of Molecular, Cellular and Developmental Biology, Yale University, New Haven, Connecticut, United States of America; 2 Reference Centre for Detection of Antimicrobial Resistance, Department of Microbiology and Infection Control, University Hospital of North Norway, Tromso, Norway; 3 Armed Forces Institute of Pathology, Washington, D. C., United States of America; Yonsei University, Republic of Korea

## Abstract

External guide sequences (EGSs) have successfully been used to inhibit expression of target genes at the post-transcriptional level in both prokaryotes and eukaryotes. We previously reported that EGS accessible and cleavable sites in the target RNAs can rapidly be identified by screening random EGS (rEGS) libraries. Here the method of screening rEGS libraries and a partial RNase T1 digestion assay were used to identify sites accessible to EGSs in the mRNA of a global virulence regulator MglB from *Francisella tularensis*, a Gram-negative pathogenic bacterium. Specific EGSs were subsequently designed and their activities in terms of the cleavage of mglB mRNA by RNase P were tested *in vitro* and *in vivo*. EGS73, EGS148, and EGS155 in both stem and M1 EGS constructs induced mglB mRNA cleavage *in vitro*. Expression of stem EGS73 and EGS155 in *Escherichia coli* resulted in significant reduction of the mglB mRNA level coded for the *F. tularensis mglB* gene inserted in those cells.

## Introduction

*Francisella tularensis* is a Gram-negative, facultative intracellular bacterium that is the etiological agent of tularemia [1]. Tularemia is primarily a disease of the Northern hemisphere that is spread by blood-sucking insects or acquired from contact with infected animals such as rabbits or rodents. In addition, *F. tularensis* is a category A agent of biowarfare/biodefense due to its high infectivity and lethality when inhaled [2]. Survival and replication inside macrophages of hosts is important for the virulence of *F. tularensis*. The MglA and MglB proteins encoded in an operon regulate transcription of a particular set of genes, some of which are required for intramacrophage growth and virulence of *F. tularensis* [2]–[6].

Inhibition of gene expression at the RNA level has been achieved by base-pairing EGSs with mRNAs of specific genes to form a complex mimicking the natural substrates of RNase P [7]–[11]. RNase P is a ubiquitous enzyme and its prime physiological role is to cleave tRNA precursors (ptRNAs) to generate the 5′ termini of mature tRNA molecules [12]. RNase P can specifically cleave any RNA *in vitro* and *in vivo*, provided that the target RNA is hybridized to an EGS that is complementary to the targeted region [13], [14]. EGSs have been designed to convert antimicrobial resistance to sensitivity [8], [11], inhibit bacterial viability, and decrease secretion of virulence factors involved in host cell invasion in Gram-negative bacteria [9], [10].

In *Escherichia coli*, RNase P consists of M1 RNA and C5 protein. The M1 RNA can execute the enzymatic function *in vitro* in the absence of the C5 protein. Depending on whether or not M1 RNA is covalently linked with an EGS, EGSs are classified as M1 EGSs and stem EGSs, respectively.

Here we describe application of stem and M1 EGSs to induce *F. tularensis* mglB mRNA cleavage by RNase P in *E. coli*. Stem and M1 EGSs targeting positions 73, 148, and 155 cleaved the mglB mRNA *in vitro*. Expression of stem EGSs targeting positions 73 and 155 resulted in a 50∼60% reduction of the mglB mRNA level in *E. coli*.

A similar successful method has been described to test EGSs on different pathogens [15].

## Results

### Mapping *in vitro* of accessible sites in the mglB mRNA

**Figure 1:**
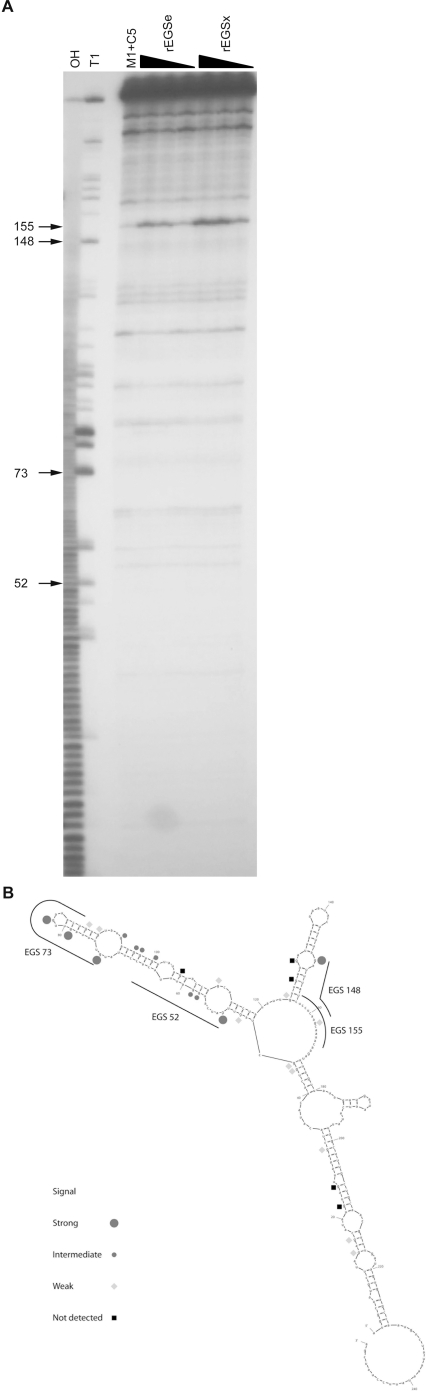
Mapping of accessible sites in the target mglB mRNA by RNase T1 and rEGS libraries. A. Polyacrylamide gel electrophoresis of cleavage products of 5′end labeled mglB mRNA generated by RNase P holoenzyme and rEGSe or rEGSx libraries. The RNase P holoenzyme was reconstituted by 10 nM M1 RNA and 100 nM C5 protein. mglB mRNA cleavage products were separated on 8% polyacrylamide/7 M urea gels together with an alkali ladder and a partial RNase T1 digest of the same RNA. Arrows indicate positions selected for further analyses. The black filled triangles represent decreasing concentrations of rEGSs (1000-, 100-, and 10-fold molar excess to the mglB mRNA) incubated with RNase P holoenzyme. Lane OH represents the alkali ladder. Lanes T1 and M1+C5 represent reaction with RNase T1 and RNase P holoenzyme, respectively. The difference between rEGSe and rEGSx is the length of 3′ end of rEGS mRNA resulted from the *Bst*NI digestion of the DNA fragments for *in vitro* transcription. The numbers indicate the positions of cleavage relative to the *mglB* translational initiation site. B. Complexes between the mglB mRNA and EGS52, EGS73, EGS148, and EGS155. The secondary structure of the partial mglB sequence used for the assay was created by the Mfold program [21]. Signal strength is according to the RNase T1 assay.

Many RNAs spontaneously self-assemble into highly folded structures. EGS accessible sites within target RNAs are determined to facilitate the design of specific EGSs. Several methods have been successfully used to identify accessible sites on various target RNAs [16], [17]. In this study, the rapid rEGS assay was used to screen for stem EGSs that selectively cleave the target mglB mRNA. An effective stem EGS candidate should meet the following requirements: an EGS-directed cleavage reaction occurs only if the target RNA is incubated with both rEGS and the RNase P holoenzyme; the amount of cleavage product increases as the amount of rEGS increases. Figure 1A suggests that an EGS targets the position 155 (Fig. 1A and 1B).

The 5′ end nucleotide of ptRNA cleavage products of RNase P is guanine (G) in most cases. The rEGSs consisting of the random 14-nucleotide sequence (N_14_) and a cytosine (C) were designed to bind to a G nucleotide in the target RNA. Interestingly the nucleotide at position 155 in the mglB RNA is a C rather than a G. In principle, the nucleotide next to the CACCA sequence in rEGS(s) might be G, which base pairs with the nucleotide C at the position 155 of the mglB sequence.

As described previously for screening rEGSs targeting the vjbR mRNA of *Brucella melitensis* [17], both rEGSe and rEGSx libraries were used (Fig. 1A). The only difference between rEGSe and rEGSx is the shorter length of the 3′ end of rEGSx mRNA that resulted from the *Bst*NI digestion of the DNA fragments for *in vitro* transcription. Consistent with previous report (17), the rEGSx library gave much stronger cleavage than the rEGSe library (Fig. 1A).

The partial RNase T1 digestion assay is normally used to locate the exposed G nucleotides in the target RNA [18]. The signal strength of bands in the T1 lane in Figure 1A indicates the accessibility of the G nucleotides in the mglB mRNA (Fig. 1A and 1B). Although not identified by the rEGS assay, positions 52, 73, and 148 could be EGS accessible sites (Fig. 1A and 1B).

### Cleavage *in vitro* of the mglB mRNA by specific EGSs

**Figure 2:**
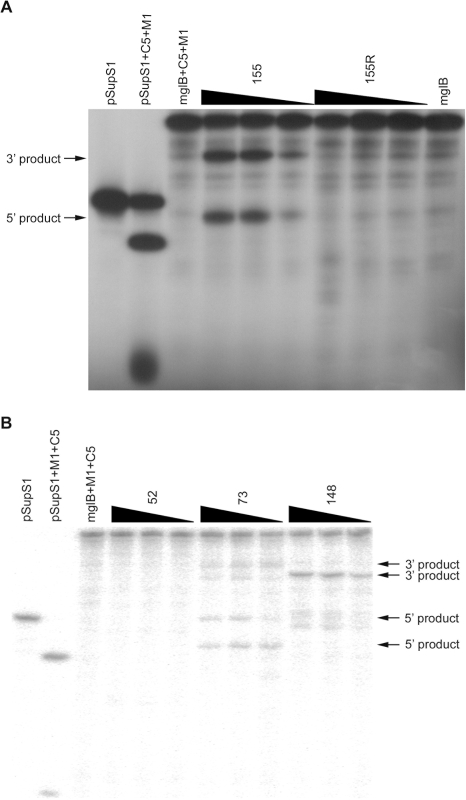
Cleavage *in vitro* of the mglB mRNA by the RNase P holoenzyme in the absence or presence of stem EGSs. A. Activities of stem EGS155 and EGS155R. B. Activities of stem EGS52, EGS73, and EGS148. If needed, 10 nM M1 RNA and 100 nM C5 protein in buffer PA were added to reconstitute the RNase P holoenzyme. The mglB mRNA cleavage products were separated on 8% polyacrylamide/7 M urea gels along with the mglB mRNA alone or the mglB mRNA with the RNase P holoenzyme. Internally labeled pSupS1 ptRNA was used as a positive control to check for activity of the reconstituted RNase P holoenzyme. EGSs were added in 100-, 50-, and 10-fold molar excess to the mglB mRNA, denoted by black triangles.

Stem EGSs and M1 EGSs targeting the potential cleavage sites, 52, 73, 148, and 155 (rEGS), were respectively made and tested in cleavage assays *in vitro*. To follow the sequence of rEGSs, N_14_CACCA, we designed EGS155R assuming the targeted position 155 to be a G nucleotide. EGS155, which perfectly base-pairs with the target mglB sequence, was also designed. Figures 2A and 2B indicate that stem EGSs targeting positions 73, 148, and 155 were able to cleave the mglB mRNA. Increase of the EGSs concentration resulted in increase of the amount of cleavage products (Fig. 2A and 2B). Failure in base-pairing of the nucleotide immediately next to ACCA in EGS155R with the target mglB sequence resulted in loss of cleavage activity (Fig. 2B). The cleavage sites were confirmed as shown in Figure 1A by 5′ end-labeling the mglB mRNA, cleavage assays *in vitro*, and then separation by denaturing PAGE with alkali-treated mglB mRNA and partial RNase T1 digested mglB mRNA as size markers (data not shown).

**Figure 3:**
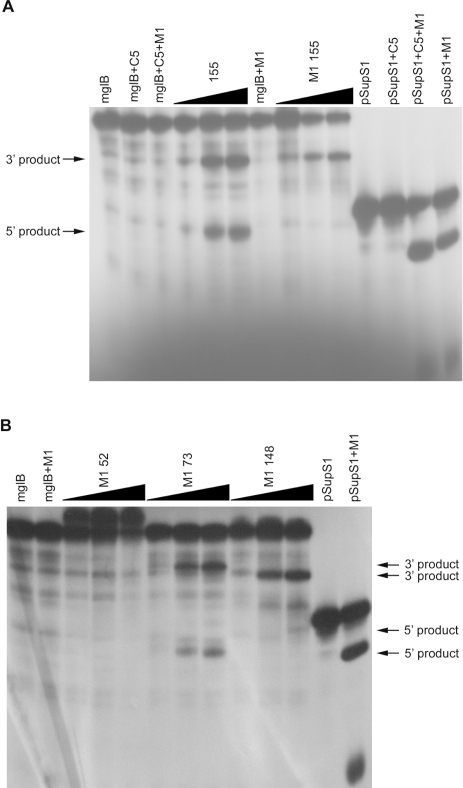
Cleavage *in vitro* of the mglB mRNA by the M1 EGSs alone in the presence of high MgCl_2_ concentration. A. Activities of M1 EGS155. B. Activities of M1 EGS52, M1 EGS73, and M1 EGS148. The RNase P holoenzyme was reconstituted by 10 nM M1 RNA and 100 nM C5 protein. If M1 EGS or M1 RNA was added in the absence of the C5 protein, additional 90 mM MgCl_2_ was added. The mglB mRNA cleavage products were separated on 8% polyacrylamide/7 M urea gels together with the mglB mRNA alone or the mglB mRNA with M1 and/or C5 components of the RNase P holoenzyme. Internally labeled pSupS1 ptRNA was used as a positive control to check for activity of the reconstituted RNase P holoenzyme or M1 RNA alone in the presence of high MgCl_2_ concentration. Stem EGS155 and M1 EGSs (M1 EGS52, M1 EGS73, M1 EGS148, or M1 EGS155) were added in 10-, 50- or 100-fold molar excess to mglB mRNA, denoted by black triangles.

Figures 3A and 3B show that higher concentration of Mg^2+^ (100 mM) induced the M1 EGSs in the absence of RNase P holoenzyme to give the same cleavage patterns as the corresponding stem EGSs in the presence of *E. coli* RNase P holoenzyme (Fig. 2A, 2B, 3A, and 3B). Consistent with the results of stem EGSs, only M1 EGSs targeting positions 73, 148, and 155 cleaved the mglB mRNA. Increase of the M1 EGSs concentration resulted in increase of the amount of cleavage products (Fig. 3A and 3B). M1 EGS52 and M1 EGS155R could not cleave the mglB mRNA (Fig. 3B and data not shown).

### Specific EGSs induce reduction of the mglB mRNA level in *E. coli*

To confirm if EGS73, EGS148, and EGS155 induce reduction of the mglB mRNA level *in vivo*, we transcribed the *mglB* gene and the stem EGSs from the same plasmid in *E. coli* by creating the plasmids pMlac-mglB-EGS73, pMlac-mglB-EGS148, pMlac-mglB-EGS155, pMlac-mglB-M1EGS73, pMlac-mglB-M1EGS148, and pMlac-mglB-M1EGS155.

**Figure 4:**
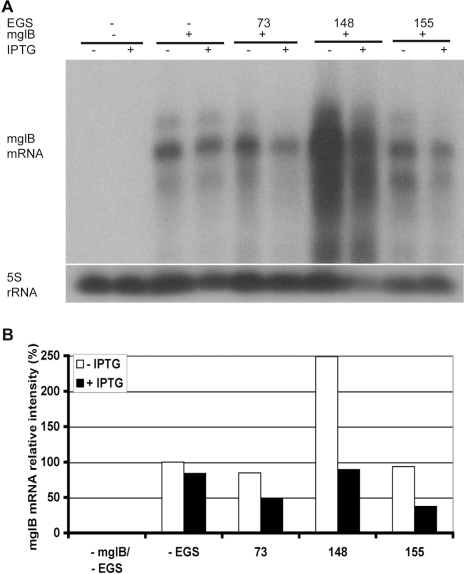
Analysis of stem and M1 EGSs activity *in vivo*. A. Northern analysis of the mglB mRNA level in *E. coli*. The plasmid vector pKB283-Mlac without (−) *mglB* nor (−) EGS was used as a negative control. The plasmid vector pMlac-mglB with (+) *mglB* but without (−) EGS was used as a positive control. The plasmids pMlac-mglB-EGS73, pMlac-mglB-EGS148, and pMlac-mglB-EGS155 carried both (+) *mglB* and EGS73, EGS148, and EGS155, respectively. Total RNAs were prepared from *E. coli* BL21(DE3) cells in the absence (−) or presence (+) of IPTG to induce expression of the EGSs listed. The RNA samples were separated on 2% agarose gels. The mglB mRNA was probed with 5′ end labeled oligonucleotide RNA155 and the 5S rRNA with 5S1 on the same membrane. The experiments were independently done twice and only a typical figure was shown. B. Average of the relative mglB mRNA level in *E. coli*. The ratios of the mglB mRNA to the 5S rRNA for cells carrying different plasmids were calculated. The ratio for cells carrying pMlac-mglB was chosen as 100% to calculate the relative mglB mRNA level in other cells listed.

Transcription of *mglB* was under the control of the *rnpB* promoter and transcription of EGSs under the control of the IPTG-inducible T7 promoter (data not shown). To avoid the mutual influence of the two promoters, mglB and EGSs were transcribed in opposite directions. Figures 4A and 4B show that IPTG-induced expression of stem EGS73 and EGS155 resulted in reduction of the mglB mRNA level to 40∼50% of that without the EGSs in *E. coli* cells. Surprisingly, the mglB mRNA level increased in the presence of leaky expression of stem EGS148 for unknown reasons but increase of EGS148 transcription still resulted in reduction of the mglB mRNA level (Fig. 4A and 4B). None of the M1EGS73, M1EGS 148, and M1EGS155 induced significant reduction of the mglB mRNA level in *E. coli* (data not shown) as shown previously for other M1 EGSs in *E. coli* (C. Guerrier-Takada, Yale University, unpublished data).

## Discussion

We successfully identified two EGSs, EGS73 and EGS155, which induced reduction of the mRNA level of the global virulence regulator MglB from *F. tularensis* both *in vitro* and in *E. coli*. In a previous report, stem EGS117 and EGS166 were identified to cleave the mRNA of a virulence regulator VjbR from *B. melitensis in vitro* [17]. We subsequently found that stem EGS117 reduced the vjbR mRNA level in *E. coli* by 80% and stem EGS166 by 100% compared with that in the absence of the two EGSs (data not shown). Thus, it is possible that if the EGSs, EGS73 and EGS155 for mglB of *F. tularensis* and EGS117 and EGS166 for vjbR of *B. melitensis*, are delivered to the pathogenic bacteria, they will reduce their target mRNA levels so that virulence of the bacteria will be reduced.

The method of screening rEGS libraries has successfully been used to identify accessible and cleavable sites in the target RNA [17]. All specific EGSs that we have designed so far based on the results of the rEGS method are able to cleave the target mRNAs [17]. The efficiency of the rEGS method is better than that of any other method that has been used, including the partial RNase T1 digestion assay. However, in some cases where we searched for rEGS, we have been unable to identify any EGS-induced cleavable site or EGS accessible sites that were identified by other methods [15], [17]. There are two ways to solve the problem. One is to combine the results of the rapid rEGS method and the partial RNase T1 digestion assay to design specific EGSs, as we did in this study. The other is to make a transcript of the target gene that is much larger than the one used in this case so that the whole landscape of the targeted gene is examined.

The nucleotide G is preferred to lie immediately next to the natural ptRNA cleavage site and base-pairs with the nucleotide C before the ACCA sequence in the 3′ end of the ptRNA cleavage product [17]. When we originally designed the rEGS libraries, we used the nucleotide C after the N_14_ sequence and before the ACCA sequence to base-pair with the nucleotide G in target RNAs [17]. Interestingly, the nucleotide C at the position 155 of the mglB sequence identified by the rEGS method does not follow this rule. However, difference in base-pairing of a single nucleotide in EGS155 and EGS155R with the nucleotide G significantly affects EGS-induced RNase P cleavage activity (Fig. 2A), suggesting the importance of base-pairing between EGSs and the nucleotide next to the cleavage site. The interaction between the nucleotide T at the position 156 of the mglB sequence and the nucleotide A next to the CACCA sequence in EGS155R may be too weak to form a mRNA-EGS complex to induce RNase P cleavage activity.

While we have outlined a safe method to test the effect of EGSs on genes from pathogenic bacteria in this report, we also note that shuttle plasmids that contain the successful EGSs have been constructed and are now in the process of being tested in the actual pathogens in BL3 facilities of the Armed Forces Insitute of Pathology. The efficiency of EGS inactivation of expression of target genes can be enhanced by putting two EGSs attacking different sites on the target mRNA on a shuttle plasmid. Indeed, two EGSs attacking the same site can also be used. Agents that can be injected into mice or administered to them by nasal inhalation will be used in these experiments. Chemically modified EGSs that can be taken up by bacteria (Avi Biopharma, Corvallis, Oregon) should be used in this respect and might be successful. Clinical application of these EGSs will rely on the results of the mouse experiments.

## Materials and Methods

### Materials

Oligonucleotides were made by the Yale synthesis facility (New Haven, CT). Restriction endonucleases, DNA polymerase I Klenow fragment, T4 polynucleotide kinase, and T4 DNA ligase were purchased from New England Biolabs (Cambridge, MA). SP6 and T7 RNA polymerases were purchased from Promega (Madison, WI). Qiaquick PCR purification kit, Qiaquick gel extraction kit, and Qiaprep spin miniprep kit were purchased from Qiagen (Chatsworth, CA). AmpliTaq DNA polymerase was purchased from Roche (Indianapolis, IN). Ultrafree-MC column was purchased from Millipore (Billerica, MA). [α-^32^P]GTP and [γ-^32^P]ATP were purchased from Amersham (Piscataway, NJ) and PerkinElmer (Boston, MA).

### Strains and growth conditions

Bacterial strains used are listed in Table 1. *E. coli* cells were grown at 37°C in Luria-Bertani (LB) broth or on LB agar. *E. coli* DH5α was routinely used for subcloning and plasmid propagation. *E. coli* BL21(DE3) was used for protein expression. Ampicillin or carbenicillin (100 μg/ml) was used for plasmid selection and maintenance in *E. coli*.

**Table 1:** Bacterial strains and plasmids.

Strains/Plasmids	Relevant characteristics	References/Source
Strains		
DH5α	F^−^ φ80dlacZΔM15 Δ(lacZYA-argF)U169 deoR recA1 endA1 hsdR17(r_k_^−^ m_k_^+^) phoA supE44 λ^−^ thi-1 gyrA96 relA1	Lab collection
BL21(DE3)	F^−^ *ompT hsdS_B_*(r_B_^−^ m_B_^−^) *gal dcm* λ(DE3)	Novagen
Plasmids		
pGEM-T	Cloning vector; Amp^r^	Promega
pGEMglB	pGEM-T with *mglB* PCR product; Amp^r^	This study
pGEΔMglB	pGEMglB with *Apa*I-*Sac*II deletion; Amp^r^	This study
pSupS1	pSP64 with tRNA^ser^ insert; Amp^r^	[22]
pUCT7/AEFRNAHHT7T	pUC19-derived cloning vector with T7 promoter and terminator, and HH sequence; Amp^r^	Guerrier-Takada C, unpublished
pUCT7mglBEGS52	pUCT7/AEFRNAHHT7T with stem EGS52 *Pst*I-*Bst*EII insert; Amp^r^	This study
pUCT7mglBEGS73	pUCT7/AEFRNAHHT7T with stem EGS73 *Pst*I-*Bst*EII insert; Amp^r^	This study
pUCT7mglBEGS148	pUCT7/AEFRNAHHT7T with stem EGS148 *Pst*I-*Bst*EII insert; Amp^r^	This study
pUCT7mglBEGS155	pUCT7/AEFRNAHHT7T with stem EGS155 *Pst*I-*Bst*EII insert; Amp^r^	This study
pUCT7mglBEGS155R	pUCT7/AEFRNAHHT7T with stem EGS155R *Pst*I-*Bst*EII insert; Amp^r^	This study
pUCT7/M1AEFRNAHHT7T	pUC19-derived cloning vector with T7 promoter and terminator, M1 and HH sequence; Amp^r^	Guerrier-Takada C, unpublished
pUCT7M1mglBEGS52	pUCT7/M1AEFRNAHHT7T with stem EGS52 *Pst*I-*Bst*EII insert; Amp^r^	This study
pUCT7M1mglBEGS73	pUCT7/M1AEFRNAHHT7T with stem EGS73 *Pst*I-*Bst*EII insert; Amp^r^	This study
pUCT7M1mglBEGS148	pUCT7/M1AEFRNAHHT7T with stem EGS148 *Pst*I-*Bst*EII insert; Amp^r^	This study
pUCT7M1mglBEGS155	pUCT7/M1AEFRNAHHT7T with stem EGS155 *Pst*I-*Bst*EII insert; Amp^r^	This study
pUCT7M1mglBEGS155R	pUCT7/M1AEFRNAHHT7T with stem EGS155R *Pst*I-*Bst*EII insert; Amp^r^	This study
pKB2835′HHC5EGS13′HHM1T	pUC19-derived cloning vector pKB283 with 5′HHC5EGS13′HH insert; Amp^r^	[18]
pKB283-mglB	pKB283 with *mglB Bam*HI-*Bst*EII insert; Amp^r^	This study
pMlac	pUC19 with a mutated *lacZ* promoter; Amp^r^	[15]
pMlac-mglB	pKB283-mglB with a mutated *lacZ* promoter; Amp^r^	This study
pKB283-Mlac	pKB2835′HHC5EGS13′HHM1T with a mutated *lacZ* promoter; Amp^r^	This study
pMlac-mglB-EGS73	pMlac-mglB with stem EGS73 *Hind*III-*Eco*RI insert; Amp^r^	This study
pMlac-mglB-EGS148	pMlac-mglB with stem EGS148 *Hind*III-*Eco*RI insert; Amp^r^	This study
pMlac-mglB-EGS155	pMlac-mglB with stem EGS155 *Hind*III-*Eco*RI insert; Amp^r^	This study
pMlac-mglB-M1EGS73	pMlac-mglB with M1 EGS73 *Hind*III-*Eco*RI insert; Amp^r^	This study
pMlac-mglB-M1EGS148	pMlac-mglB with M1 EGS148 *Hind*III-*Eco*RI insert; Amp^r^	This study
pMlac-mglB-M1EGS155	pMlac-mglB with M1 EGS155 *Hind*III-*Eco*RI insert; Amp^r^	This study

Amp^r^, ampicillin resistant; HH, hammerhead.

### Preparation of the target mglB mRNA

Restricted genomic DNA of *F. tularensis* was obtained from the Armed Forced Institute of Pathology, Washington, DC. The template for the mglB mRNA of *F. tularensis* was prepared as described previously [17]. To map accessible sites in the mglB mRNA, the 5′ fragment was amplified by PCR using primers mglB F (5′- ATGGCTATGCTTAGAGCATATG-3′) and mglB R (5′- TCATCATCTTCGCCTTCATT-3′). The PCR fragment was sub-cloned into the pGEM-T vector, generating the plasmid pGEMglB. To reduce the length of the vector sequence between the T7 promoter in the pGEM-T vector and the inserted DNA fragment, the plasmid pGEMglB was digested with the restriction enzymes *Apa*I and *Sac*II, purified from an agarose gel using Ultrafree-MC columns, blunt ended using DNA polymerase I Klenow fragment, and then re-ligated using T4 DNA ligase. The resulting plasmid pGEΔMglB was digested with the restriction enzymes *Fok*I and *Taq*I, ethanol-precipitated and dissolved in distilled water, and used as the template for *in vitro* transcription using T7 RNA polymerase [17]. For internally labeled transcripts, [α-^32^P]GTP was used during the transcription reaction. For the mapping of cleavage sites, transcripts were labeled at the 5′ end using [γ-^32^P]ATP and T4 polynucleotide kinase.

### Mapping of accessible sites in the mglB mRNA

The rEGS assay for the mglB mRNA was performed as described previously [17]. Partial RNase T1 reaction was performed as previously described (8). After the reaction, the mRNA was separated on an 8% polyacrylamide sequencing gel that contained 7 M urea. Partial alkaline ladder digests were carried out as previously described [17]. Cleavage sites were determined by comparing rEGS-mediated and partial RNase T1 cleavage products to partial alkaline ladder results. The yeast suppressor precursor tRNA^Ser^ (SupS1) was internally labeled with [α-^32^P]GTP and used as a substrate in control reaction [15], [19].

### Preparation of stem and M1 EGSs against the mglB mRNA

Specific EGSs were designed based on the rEGS assay and partial RNase T1 assay according to previous reports [17], [18]. The specific EGSs used for *in vitro* cleavage assays were phosphorylated at their 5′ ends, annealed together by heating at 95°C for 5 min and cooled down slowly. The annealed oligos had the restriction enzymes *Pst*I and *Bst*EII overhangs at the 5′ and 3′ ends, respectively. These oligos used for EGS52, EGS73, EGS148, EGS155, and EGS155R were 52 (5′- GTGTAAATCCATGATCACCAG-3′), 52 compl (5′- GTGACCTGGTGATCATGGATTTACACTGCA-3′), 73 (5′- GAGTATCAACTAAAACACCAG-3′), 73 compl (5′- GTGACCTGGTGTTTTAGTTGATACTCTGCA-3′), 148 (5′- GTTGAGGAGATAAATCACCAG-3′) , 148 compl (5′- GTGACCTGGTGATTTATCTCCTCAACTGCA-3′), 155 (5′- GGTATTGCTTGAGGAGACCAG-3′), 155 compl (5′- GTGACCTGGTCTCCTCAAGCAATACCTGCA-3′), 155R (5′- GGTATTGCTTGAGGACACCAG-3′), 155R compl (5′- GTGACCTGGTGTCCTCAAGCAATACCTGCA-3′). The hybridized EGS oligos were inserted into a pUC19-based vector containing the T7 promoter, pUCT7/AEFRNAHHT7T, pre-digested with *Pst*I and *Bst*EII, generating the plasmids pUCT7mglBEGS52, pUCT7mglBEGS73, pUCT7mglBEGS148, pUCT7mglBEGS155, and pUCT7mglBEGS155R. To create the templates for M1 EGSs, the hybridized EGS oligos were inserted into a pUC19-based vector containing the T7 promoter and an M1, pUCT7/M1AEFRNAHHT7T, pre-digested with *Pst*I and *Bst*EII, generating the plasmids pUCT7M1mglBEGS52, pUCT7M1mglBEGS73, pUCT7M1mglBEGS148, pUCT7M1mglBEGS155, and pUCT7M1mglBEGS155R. These constructs were sequenced and the plasmids digested with *Bst*EII, ethanol-precipitated and dissolved in distilled water, and used as templates for *in vitro* transcription.

### EGS activity assays *in vitro*

EGS activity assays were performed according to a previous report [17]. The EGS in 100-, 50- or 10-fold molar excess was incubated with 10 nM of 5′ end-labeled or internally labeled target mRNAs in PA buffer (50 mM Tris-Cl, pH 7.5, 10 mM MgCl_2_, 100 mM NH_4_Cl) for 5 min at room temperature. Subsequently, 10 nM of M1 RNA and 100 nM of C5 protein were added to the mixture and further incubated for 30 min at 37°C. For reactions with M1 or M1 EGS RNA alone, 100 nM of M1 or M1 EGS RNA was used in PA100 buffer (PA buffer containing 100 mM MgCl_2_). Reactions in 10 μl volume were performed at 37°C for 30 min, and terminated by adding 10 μl of 10 M urea/10% phenol solution. The product was separated on 5% polyacrylamide that contained 7 M urea gels.

### Construction of plasmids for the EGS activity assay in *E. coli*

The plasmids contain the platform expressing both a target mRNA and a specific EGS by using the *rnpB* promoter for the target mglB mRNA and the T7 promoter for various EGSs. An additional ‘C’ was introduced into the -35 region of *lacZ* on the plasmids in order to inactivate transcription from the *lacZ* promoter by making a *Stu*I site [15].

The full-length *mglB* gene was amplified by PCR using primers mglBtot F (5′- CAGGATCCAAAAGGAGCTTAATATGGCTATG-3′) and mglBtot R (5′- CACAGGTGACCGAATTAATCTAACGAAAGAACG-3′), containing restriction sites for *Bam*HI and *Bst*EII, respectively. The PCR products was digested with *Bam*HI and *Bst*EII, purified from agarose gel using Ultrafree-MC columns, and cloned into a pUC19-based plasmid vector, pKB2835′HHC5EGS13′HHM1T, digested with the same restriction enzymes, generating the plasmid pKB283-mglB.

To make the mutagenesis in the -35 region of *lacZ* in the plasmids, the *Hind*III-*Afl*III fragment, which carries the mutated *lacZ* promoter, from the plasmid pMlac was cut and inserted into the plasmid pKB283-mglB to create pMlac-mglB. The *Hind*III-*Afl*III fragment was also inserted into the plasmid pKB2835′HHC5EGS13′HHM1T to create pKB283-Mlac.

The *Hind*III-*Eco*RI fragments from the plasmids pUCT7mglBEGS73, pUCT7mglBEGS148, pUCT7mglBEGS155, pUCT7M1mglBEGS73, pUCT7M1mglBEGS148, and pUCT7M1mglBEGS155 were blunt-ended with the Klenow fragment and inserted into the pMlac-mglB plasmid which was cut with *Eco*RI, and then blunt-ended with the Klenow fragment. The resultant plasmids were named pMlac-mglB-EGS73, pMlac-mglB-EGS148, pMlac-mglB-EGS155, pMlac-mglB-M1EGS73, pMlac-mglB-M1EGS148, and pMlac-mglB-M1EGS155.

### Northern analysis

Freshly transformed cells were used for Northern analysis. BL21(DE3) competent cells (150 μl) were mixed with 0.1 μg of the appropriate plasmid and transformed at 42°C for 90 sec. The transformed cells were cultured overnight at 37°C on an LB plate with ampicillin. It is essential to use freshly transformed cells: when the cells were cultured from the glycerol stock of transformed cells, the expression of EGS was poor very often. A colony was picked from the LB plate and was cultured overnight in LB media containing carbenicillin at 37°C.

The overnight culture was diluted 1∶100 and incubated at 37°C to OD600 = 0.6. The cells were split into two tubes and 2 mM (final concentration) IPTG was added into one of the tubes. The cells were further incubated for one hour and total RNA was prepared as described previously [20]. The RNAs (4 μg) were separated on a 2% agarose gel.

Northern blot analysis was performed as described previously [7]. Oligonucleotides of RNA155 (5′-CTT GTA TTG CTT GAG GAG ATA AAT CT-3′), 73 compl, 148 compl, 155 compl, and 5S1 (5′-TAC CAT CGG CGC TAC GGC GTT TCA CTT C-3′) were labeled at their 5′ ends using [γ-^32^P]ATP and then used as probes for mglB mRNA, EGS73, EGS148, EGS155, and 5S rRNA, respectively.
